# Astrocyte heterogeneity and interactions with local neural circuits

**DOI:** 10.1042/EBC20220136

**Published:** 2023-03-03

**Authors:** Matthew G. Holt

**Affiliations:** Instituto de Investigação e Inovação em Saúde (i3S), University of Porto, 4200-135 Porto, Portugal

**Keywords:** astrocytes, heterogeneity, neural circuits, peripheral process, synapses

## Abstract

Astrocytes are ubiquitous within the central nervous system (CNS). These cells possess many individual processes which extend out into the neuropil, where they interact with a variety of other cell types, including neurons at synapses. Astrocytes are now known to be active players in all aspects of the synaptic life cycle, including synapse formation and elimination, synapse maturation, maintenance of synaptic homeostasis and modulation of synaptic transmission. Traditionally, astrocytes have been studied as a homogeneous group of cells. However, recent studies have uncovered a surprising degree of heterogeneity in their development and function, suggesting that astrocytes may be matched to neurons to support local circuits. Hence, a better understanding of astrocyte heterogeneity and its implications are needed to understand brain function.

## Introduction

Astrocytes comprise the largest class of glial cells in the mammalian brain and are essential for central nervous system development and function [1]. Differences between astrocytes have been reported. Classically, astrocytes have been classified based on gross morphology into protoplasmic (gray matter) astrocytes and fibrous (white matter) astrocytes. In addition, there is known variability in the expression of marker proteins, such as glial fibrially acidic protein (GFAP) and excitatory amino acid transporter 1 (EAAT1/GLAST), as well as in astrocyte numbers across brain regions. Finally, various types of specialist astrocytes have been reported in retina (Müller glia) and cerebellum (Bergmann glia). Despite these obvious differences, however, astrocytes have traditionally been viewed as a largely homogeneous cell type [2].

Recent advances in tools to specifically label and manipulate astrocytes are beginning to change this view [3]. It is now widely acknowledged that astrocytes interact with a wide variety of neuronal types [4,5] and are key players at all stages of the synaptic life cycle, including synapse formation and elimination, synapse maturation, maintenance of synaptic homeostasis and modulation of synaptic transmission [6]. This diversity of functions raises the rather obvious question of whether they are performed by all astrocytes, as presupposed, or whether functional subsets of astrocytes exist.

**Figure 1:**
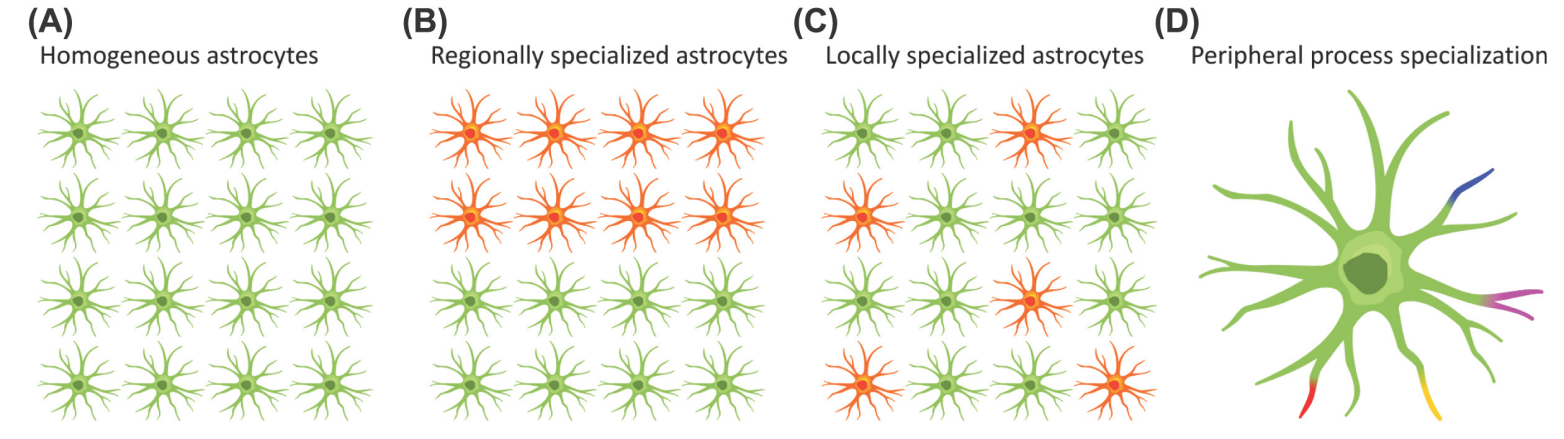
Possible degrees of astrocyte heterogeneity Different levels of astrocyte heterogeneity have been proposed, including (**A**) homogeneous astrocytes – astrocytes are essentially identical throughout the brain, (**B**) astrocytes show heterogeneity between brain regions, (**C**) astrocytes show heterogeneity within brain regions, and (**D**) subcellular specialization – astrocytes show heterogeneity at the level of their individual processes (or even within an individual process). Mounting evidence now favors heterogeneity at the subcellular level, with peripheral astrocyte processes, which act as local signaling domains, responding to local neuronal activity. Modified from [112].

Evidence is now accumulating that astrocytes show profound differences in developmental origin, molecular profile, physiology and functional outputs, which combine to produce a previously unsuspected level of heterogeneity both between and within brain regions. The goal of this mini-review is 3-fold. First, I aim to discuss recent advances in our understanding of astrocyte heterogeneity: to do this, I will concentrate the discussion on protoplasmic astrocytes, principally in mouse cortex, where we arguably have the most complete picture of the astrocyte life-cycle from birth through to adulthood – although other examples will be used when instructive. Readers interested in other astrocyte types are, therefore, directed to recent review articles, which cover aspects of their development and function [7,8]. Second, I aim to discuss the evidence for a central role of neurons in determining local (protoplasmic) astrocyte identity as a means to ensure optimal local circuit formation and/or function. Finally, as astrocyte processes are in direct with synapses, I advance the idea that subcellular compartmentalization is likely a principal driver of (functional) heterogeneity (Figure 1).

Given the short-format of this article, readers interested in a comprehensive review of the literature relating to aspects of protoplasmic astrocyte development, form or function are pointed towards several recent articles, each of which covers a specific topic in depth [2,9–12].

## Development

The production of neurons and astrocytes during development is intimately connected and (arguably) best understood in the mouse cortex. Its basic anatomy is generally defined based on the anatomical arrangement of excitatory neurons into six horizontal layers and numerous vertical columns, which form the basis of cortical microcircuits [13]. The generation of cortical neurons begins at approximately embryonic (E) day 10–11. Regional patterning events generate progenitor cells (radial glia) in the ventricular zone. During mid-to-late embryonic development, radial glia generate the vast majority of cortical excitatory neurons. As radial glia extend long processes which span the cortex, these cells also acts as guides for migrating neurons, explaining how the cortex is built in an ‘inside-out’ fashion (in contrast, cortical interneurons are generated outside of the developing cortex in the subpallium and migrate tangentially over long distances into the cortex) [13].

**Figure 2:**
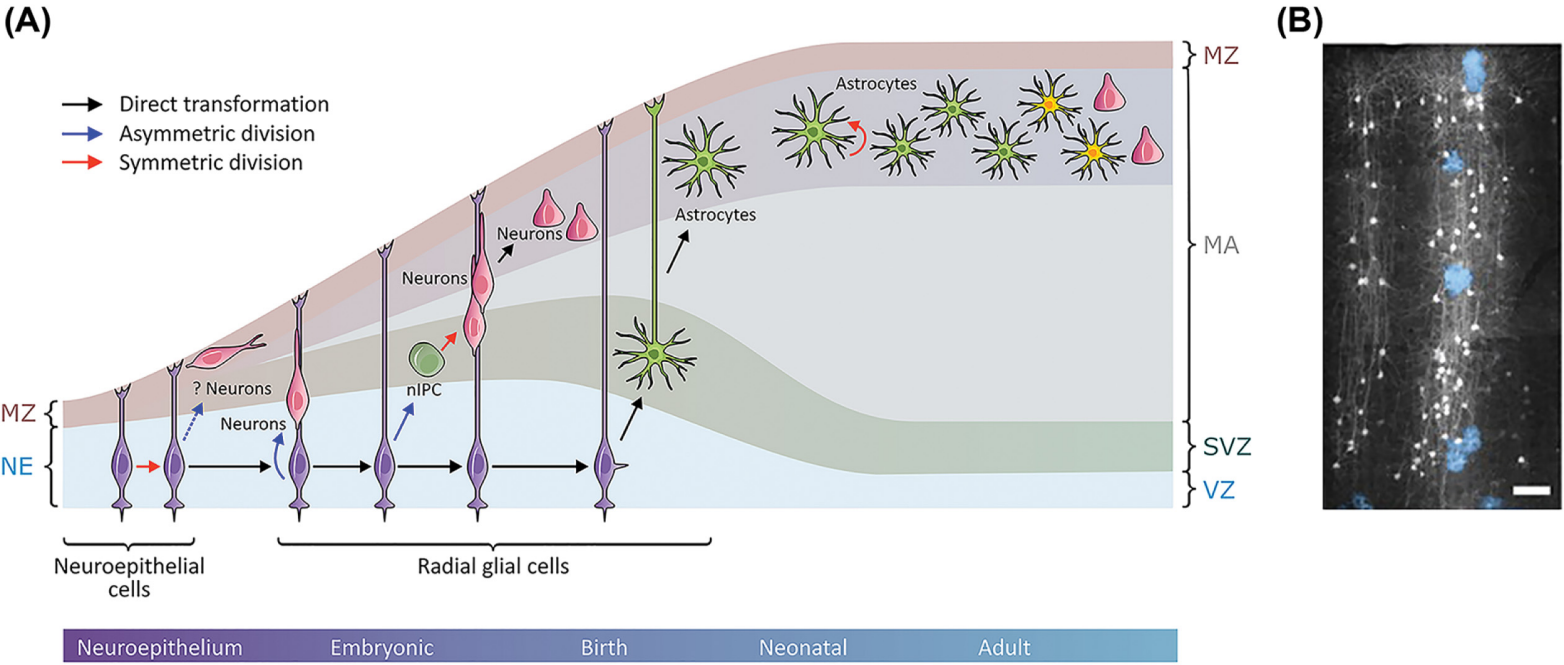
Coincident generation of neurons and protoplasmic astrocytes from radial glia (**A**) Schematic illustrating the relationship between neuro- and gliogenesis in the developing mouse cortex. Progression from the embryo to adulthood is shown left to right. Early in embryogenesis, neuroepithelial cells divide, generating additional neuroepithelial cells and potentially some neurons. As the brain develops, neuroepithelial cells elongate and transform into radial glia. Radial glia cells act as the principal source of neurons in the developing cortex, either through asymmetric division to generate neurons directly, or via intermediate progenitor cells (nIPC). Note that radial glia extend from the ventricle surface through the developing cortex, acting as a guide for neuronal migration. In late embryogenesis, most radial glia detach from the ventricle surface and generate a limited population of astrocytes. This astrocyte population expands by local proliferation, with resulting progeny tiling the parenchyma in a largely non-overlapping manner. Various aspects of astrocyte identity in the adult cortex appear dependent on interactions with local neurons (indicated with yellow astrocytes). Solid arrows are supported by experimental evidence; dashed arrows are hypothetical; MA, mantle; MZ, marginal zone; NE, neuroepithelium; SVZ, subventricular zone; VZ, ventricular zone. Based on [113] and [25]. (**B**) Reporter mice, expressing green fluorescent protein (GFP) in a subset of progenitors, develop cortical columns containing both neurons and astrocytes, derived from a common cell. Astrocytes are marked in blue for clarity; scale bar: 50 µm. Taken from [22].

**Figure 3:**
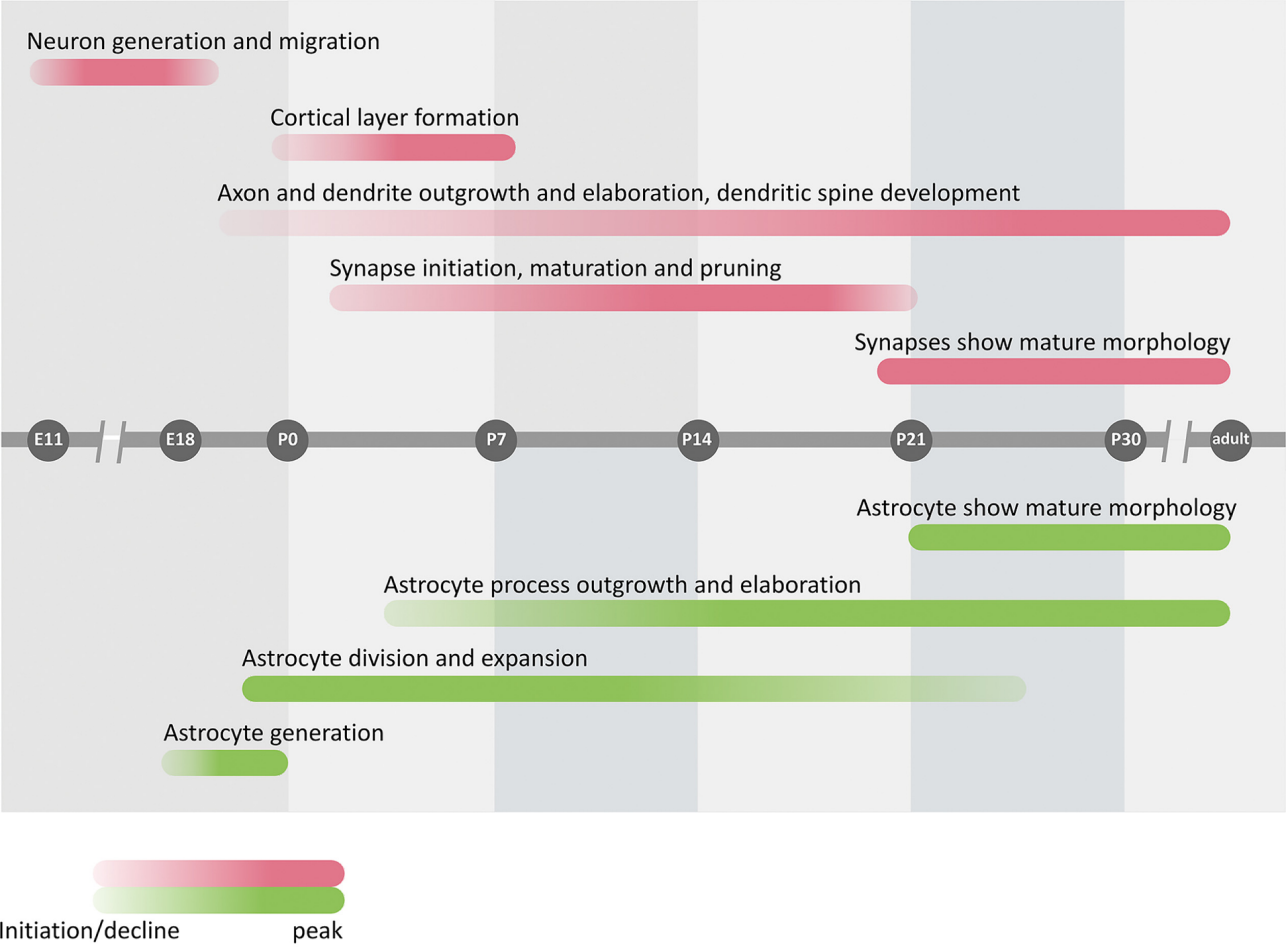
Neural circuit formation depends on the synchronized activities of neurons and astrocytes Timeline (gray) of key developmental processes in the CNS leading to synapse maturation and stabilization. Neuronal contributions (red, above) and astrocytic contributions (green, below) are shown. Each independent process is represented as a colored bar, with the gradient of color intensity marking the beginning, peak and end of the process. Modified from [13].

In cortex, radial glia switch to the production of astrocytes (the ‘gliogenic’ switch) around E18. The events underlying the gliogenic switch are incompletely understood. However, it appears that neuronal feedback may play a key role in promoting local astrocyte formation. Both neuron-committed intermediate progenitors and young neurons express the Notch ligands Jagged 1 and Delta-like 1, which activate Notch signaling in radial glia promoting astrogenesis through promoter demethylation, allowing cell type specific gene expression [14]. Cytokine release by neurons (particularly members of the interleukin 6 family) has also been proposed to stimulate gliogenesis, through stimulation of the JAK-STAT signaling pathway [15]. However, it appears that radial glia have a limited capacity for astrocyte generation, having generally disappeared by birth, when only a few astrocytes can be found in the cortex [16]. Expansion of the astrocyte population to populate the entire cortex is thought to arise from extensive local proliferation in the first few weeks of post-natal life [17] (Figure 2). Although neurons appear during embryonic development, assembly and functional maturation of synapses is concomitant to this astrocyte proliferation and maturation [13] (Figure 3).

It appears increasingly likely that this close ontological relationship between radial glia, neurons and astrocytes has important consequences for the formation of cortical microcircuits. It is now recognized that diversity in the progenitor pool has an important influence on the final identity of neurons [18,19]. Furthermore, it appears that clones of related neurons not only form a precise columnar architecture within the cortex but preferentially develop synapses among themselves [20]. The coincident generation of astrocytes from the same progenitors and their close association with sibling neurons to form a stable clonal unit [21–23], suggests a process whereby neurons act as an ‘instructional’ scaffold which ‘sculpts’ astrocytes to support the formation, maintenance and function of specific synapses within distinct local microcircuits, which form the basis of information processing in the CNS.

## Morphology

**Figure 4:**
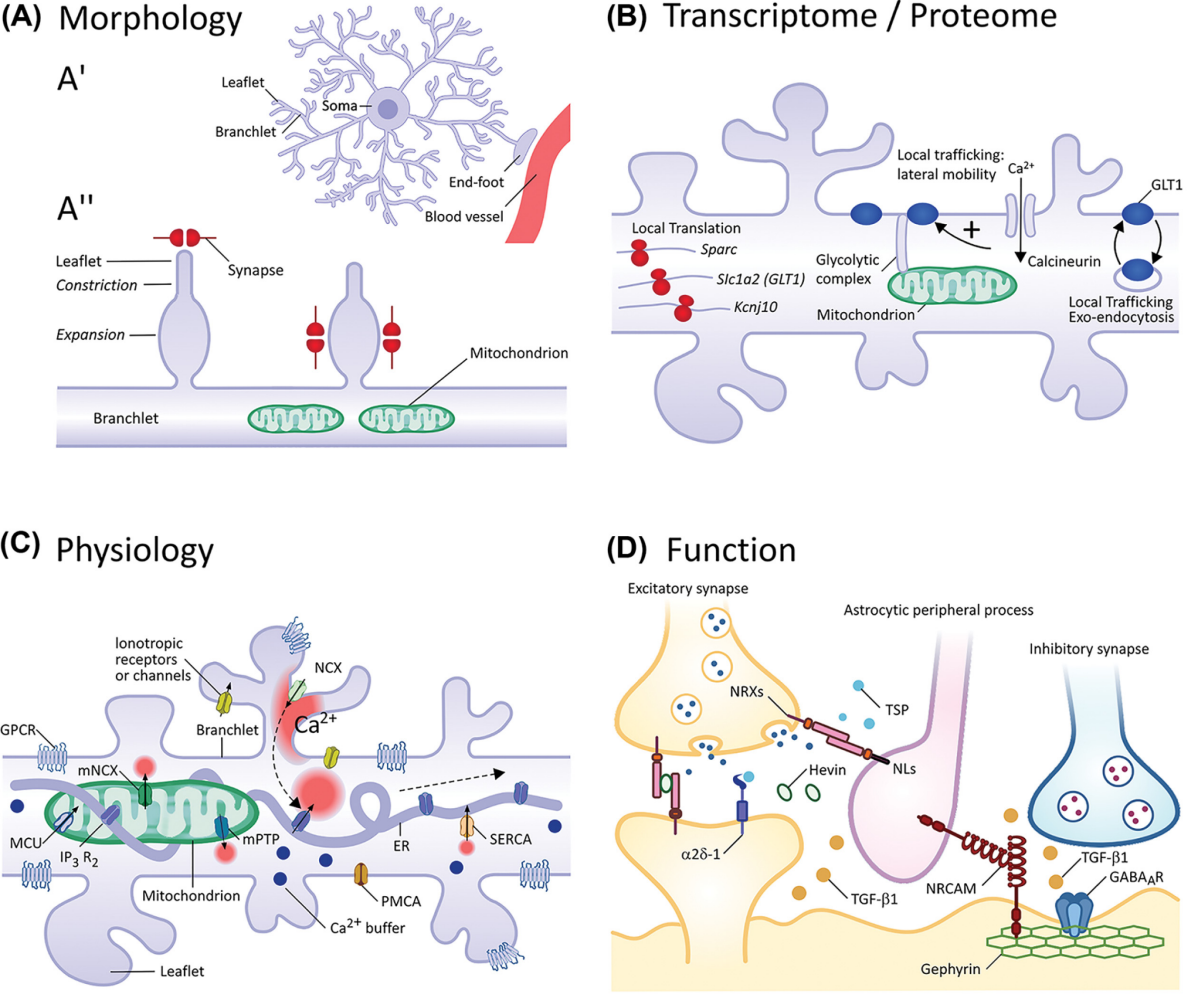
Peripheral astrocyte processes as key players in specialized astrocyte–neuron interactions Heterogeneity in adult astrocytes exists at many levels, including the morphological, transcriptomic/proteomic, physiological and functional. A significant proportion of heterogeneity may exist in the peripheral processes – as illustrated using specific examples discussed in the main text. Based on the hypothesis that astrocyte identity is highly plastic and is shaped by interactions with an ‘instructive neuronal scaffold’, specialization of peripheral processes, which mediate astrocyte–neuron interactions, seems logical and would allow astrocytes to respond quickly to changes in local synaptic activity. (**A**) Morphology – (A’) Schematic outlining basic astrocyte structure, which consists of a central soma and four to ten primary branches, which in turn branch off creating many higher order branchlets and terminal leaflets: (A’’) At the ultrastructural level, peripheral processes are comprised of both expanded and constricted segments, and often end with a constricted tip. Classically, astrocyte processes are thought to enwrap synapses at the process tip, forming a tripartite structure. However, astrocytes and synapses can also interact *en passant*, usually on so-called process expansions. Clusters of synapses tend to form at these structures, which are often characterized by the accumulation of mitochondria. (**B**) Transcriptome/Proteome – Translation of mRNAs by ribosomes (red structures) in processes will change the local proteome. Changes in protein distribution may also affect function. Local recycling of the glutamate transporter GLT1 (blue) will affect cell surface levels: Increased lateral mobility, due to the release of GLT1 from synaptically located protein scaffolds (triggered by calcineurin activity +), will control cell surface distribution. (**C**) Physiology – Intracellular Ca^2+^ increases are thought to occur through a variety of mechanisms: GPCR, G-protein coupled receptor; IP_3_R_2_, Type 2 inositol 1,4,5-trisphosphate receptor; MCU, Mitochondrial Ca^2+^ uniporter; (m)NCX, (Mitochondrial) Na^+^/Ca^2+^ exchanger; mPTP, Mitochondrial permeability transition pore; PMCA, Plasma membrane Ca^2+^ ATPase; SERCA, Sarcoplasmic/endoplasmic reticulum Ca^2+^ ATPase. (**D**) Function – Heterogeneity may underlie differential synapse formation and function through various cell–cell interactions (mediated by membrane proteins or secreted factors): α2δ-1,Voltage-dependent calcium channel subunit α2δ-1; NLs, Neuroligins; NRCAM, Neuronal cell adhesion molecule; NRXs, Neurexins; TGF-β1, Transforming growth factor-β1; TSP, Thrombospondin. (**A**) and (**C**) are modified from [11]; (**D**) is modified from [114].

Protoplasmic astrocytes have an instantly recognizable morphology, consisting of a central soma, and four to ten primary branches, which in turn branch off creating many higher order branchlets and terminal leaflets (Figure 4A’) [24]. (The specialized end-foot structure, which is responsible for astrocyte interactions with blood vessels, will not be considered in this review). This branching is paralleled by a progressive decrease in size from the µm (soma) to nm (leaflets) size domain. Astrocyte morphology appears to develop within the first three post-natal weeks [25], concomitant to synaptogenesis (Figure 3), and appears heavily dependent on a complex interplay between physical interactions with neurons [26] and the effects of soluble neurotransmitters [27].

Mature astrocytes display a variety of morphologies. A systematic study of mouse hippocampal and striatal astrocytes revealed that while these cells have equal somatic volumes, numbers of processes and cell volumes, striatal astrocytes generally occupy a larger tissue area than hippocampal astrocytes [28]. While striatal astrocyte territories typically contain many neuronal cells bodies compared with hippocampal astrocytes, the latter contain more excitatory synapses, implying distinct regional differences in how astrocytes interact with local neuronal circuits [28]. Morphological heterogeneity even extends to astrocytes occupying the same brain region. For example, cortical protoplasmic astrocytes were found to display four prominent morphological subtypes, which distribute in differing proportions through the various cortical layers [29], which may reflect differences in local neuronal activity [30]. Likewise, astrocytes in the dentate gyrus (DG) have also been shown to display morphological heterogeneity linked to DG layering [31].

Astrocyte peripheral processes have received considerable attention as their terminal ends are thought to interact with neurons – creating a structure widely referred to as the ‘tripartite synapse’. Although the degree of this interaction between astrocytes and neurons varies between brain regions (77% of synaptic interfaces in striatum, 86% in hippocampus, 90% in cortex [28,32]), this structure is obviously remarkably common. An elegant electron microscopy study has recently reconstructed astrocytes in mouse somatosensory cortex with unprecedented resolution, revealing an elaborate heterogeneity in process nanostructure [33]. In this study, processes appear as branches of undulating thickness (comprising both expanded and constricted segments), often ending with a constricted tip that is physically associated with a synapse. However, a number of connections were also apparent within the process structure, creating *en-passant* synapses: it is for this reason that the term ‘peripheral process' is likely to be more accurate than ’perisynaptic process’, which strongly implies a tripartite structure formed by a terminal process ensheathing a synapse [34,35]. Furthermore, synapses around astrocytes tend to organize into clusters of between 4 and 55 structures, with mitochondria positioned closer to larger synaptic clusters, presumably to match the high energetic demands of synaptic transmission (Figure 4A’’). Interestingly, in this study, astrocyte processes were rarely seen looping back on themselves [33]. Hence, astrocytes were proposed to possess a structure largely reminiscent of a ‘thorny bush’, rather than a ‘sponge’. This is in direct contradiction with two other recent studies of astrocyte morphology, one of which also used electron microscopy [36], while the other used super-resolution stimulated emission depletion (STED) microscopy (albeit on live rather than fixed tissue) [37]. Both these studies reported ‘frequent’ loop type structures – although, crucially, neither study quantified the frequency at which these structures appear. At present, the most parsimonious explanation for the discrepancy between these studies is the relatively small number of somatosensory astrocytes reconstructed by Salmon and colleagues [33], combined with potential morphological variability both within and between brain regions (see above) [28,29,31] – although methodological differences cannot be completely discounted at this stage. Nevertheless, the general consensus appears to be that astrocyte processes are much more structurally heterogeneous than previous thought, and this is likely to support specialized interactions with local neuronal circuits.

At present, the degree to which tripartite synapses can be remodeled (if at all) remains unclear. Historically, there is extremely strong evidence for extensive structural remodeling of synapses in brain regions exhibiting powerful biological rhythmicity, such as circadian time setting in the suprachiasmatic nucleus [38], or those involved in modulating responses during extreme physiological conditions, such as the hypothalamus in pregnancy and lactation [39]. However, the situation in other brain regions, under normal physiological conditions, remains unclear. Experiments using a Förster resonance energy transfer (FRET)-based system to measure the distance between astrocyte peripheral processes and neurons in acute slices of striatal tissue suggest that interactions between the two cell types are stable [40]. In contrast, imaging of fluorescently labeled astrocytes and neurons, both in slices and *in vivo*, suggests that remodeling of peripheral processes can occur rapidly in response to local neuronal simulation in mouse hippocampus and somatosensory cortex [41,42], with process withdrawal impacting directly on local synaptic transmission [43]. The reasons for this discrepancy are unclear. It may reflect intrinsic differences in the biology of the striatum compared to hippocampus and cortex, or it may relate to differences in the methodologies used (or a combination of both factors).

However, what is clear is that dynamic process remodeling will involve extensive modifications, presumably driven by the actin cytoskeleton [44], and will result in the redistribution of essential proteins, protein signaling complexes and mitochondria. Processes with distinct morphologies and signaling machinery may respond differently to synaptic activity. Hence, many questions remain as to how key machinery is positioned within individual processes during various states of cell activity – yet this will undoubtedly be a factor in determining functional output and influence on local circuits.

## Transcriptome/proteome

Our understanding of astrocyte heterogeneity has been massively accelerated by sequencing-based approaches. Droplet-based single cell techniques (allied to relatively shallow sequencing) [45,46] and translating ribosome affinity purification (TRAP)-based experiments [28,47,48] have been performed to compare astrocyte transcriptomes across brain regions. The results clearly demonstrate that this cell type is much more molecularly diverse than previously thought and appears to change with age [48,49]. Although the degree to which transcriptome accurately reflects the cellular proteome is unclear [28,50], a systematic comparison of hippocampal and striatal astrocytes suggests that transcriptomic diversity may well be reflected at the protein level [28].

These groundbreaking studies have since been augmented by work clearly indicating regional substructure in cortex. This can have important consequences for local circuit formation and function, as demonstrated by the work of Miller and colleagues [51]. Using a 8.3 kb fragment of the excitatory amino acid transporter 2 (EAAT2/GLT1) promoter to drive TdTomato expression resulted in a subset of astrocytes in the region of cortical layer 5 being labeled. Crucially, this subset of astrocytes was found to express and secrete the protein Norrin, which regulates local dendrite growth and spine formation [51]. Mutations in the *Ndp* gene encoding Norrin lead to Norrie disease, which is characterized by retinal abnormalities, intellectual disabilities and behavioral abnormalities, consistent with a central role for the protein in synaptogenesis [52]. This was soon followed by a study which used large-scale single molecule fluorescence *in situ* hybridization (smFISH) [53] to map single cell transcriptome data obtained using a SMART-seq2-based protocol optimized for use with astrocytes [54]. This approach revealed cortical layering of astrocytes distinct from traditional neuronal laminae. These astrocytic laminae were defined by specific patterns of gene expression, including genes involved in synapse formation and maturation, such as *Chrdl1*, among others [53]. A similar layering of molecularly distinct astrocytes in DG [31], and the differential expression of µ-crystallin along the dorsal–ventral axis in striatal astrocytes, suggests regional substructure may be a more widespread phenomenon and not restricted to cortex [28].

Hence, current work seems to point strongly to subregional specialization of astrocytes to support local circuits. In this respect, a crucial question is how astrocyte diversity is achieved in the adult brain. Recent work suggests that the pool of developmental progenitors is limited, with astrocytes passing through various immature developmental stages after exiting the cell cycle, before becoming fully mature in the adult brain [55]. There is strong evidence that maturation and diversification of adult astrocytes is strongly dependent on local neuronal activity (Figure 2). Gross manipulations which invert neuronal layering in the cortex result in aberrant astrocyte layering [29,53]. More subtle manipulations which influence local signaling have also been shown to have profound effects. Neuronal activity is synonymous with neurotransmitter release, and work from multiple groups has shown that cortical glutamate release regulates astrocyte maturation, including the acquisition of molecular identity [27,56,57]. In addition to glutamate, other signaling molecules appear capable of regulating astrocyte gene expression. Most prominent among these is sonic hedgehog (SHH). In a seminal study, Farmer and colleagues demonstrated that neurons control Bergmann glia diversity through SHH release in the cerebellum, as well as the acquisition of astrocyte identity in hippocampus [58]. Interestingly, SHH is also produced in adult mouse cortex and SHH receptors are expressed on a proportion of cortical astrocytes [59], an arrangement which appears necessary for the expression of layer-specific astrocyte genes essential for correct synapse development [60,61]. Of course, this does not preclude a contribution of other signaling pathways, such as fibroblast growth factor (FGF) signaling [62]. This idea that neurons provide an ‘instructional scaffold’ for astrocytes is made more attractive by the recent finding that in visual cortex, neuronal identity and function is refined post-natally, in an activity dependent manner [30]. Thus, it appears that neurons and astrocytes may mature into a function circuit together.

To date, the issue of molecular identity has generally been discussed at the whole cell level. However, recent work, combining use of astrocyte-specific TRAP with subcellular fractionation, has allowed the purification of ribosomes from astrocyte peripheral processes associated with synapses (synaptosomes), strongly suggesting the possibility of local translation in astrocytes [63]. Furthermore, this local translatome appears to be dynamic and capable of modification by neuronal activity [64,65]. In mice, translation of new proteins appears to drive, at least in part, the proteomic changes that occur in peripheral astrocyte processes following exposure to a fear conditioning paradigm [65], with the resultant behavioral modifications presumably arising due to changes in astrocyte–neuron interactions which impact circuit activity. In addition, local protein trafficking appears to play a role in defining the composition of the astrocyte process, in response to neuronal activity. In mature cells, the glutamate transporter GLT1 is trafficked to the cell surface and is concentrated at synaptic sites [66], in order to terminate synaptic transmission. As the transport activity of GLT1 is slow and the number of GLT1 molecules present at the synapse sterically limited, it is thought that active glutamate uptake needs to be maintained either by the rapid resupply of ‘fresh’ transporters to the plasma membrane via local recycling [67], or by the lateral exchange of GLT1 transporters from a membrane-resident pool [66]. Interestingly, astrocytes in primates and humans display increased morphological complexity compared with rodent astrocytes, with interlaminar astrocytes extending long peripheral processes which span cortical layers [68], which would further support the concept of local signaling mechanisms as a means of ensuring efficient local function (Figure 4B).

The underlying message appears to be that astrocytes are plastic and respond to their local environment. Such changes can even be deleterious, as in human astrocytes associated with tumor margins, which downregulate genes involved in synaptic function, suggesting the involvement of peritumor astrocytes in tumor-associated neural circuit dysfunction [69]. At the extreme, it appears that striatal astrocytes may be so plastic that they are able to engage a latent neurogenic program following injury [70]. Such plasticity does not appear limited to mammalian glia. Model organisms such as *Caenorhabditis* *elegans, Drosophila* and Zebrafish all appear to possess astrocyte-like cells [12]. In the case of *Drosophila*, astrocytes also appear extremely plastic [71]. The power of the genetic toolbox available in *Drosophila*, allowing simultaneous labeling and manipulation of astrocytes and their local neurons, suggests that such model organisms may provide highly useful insights into the mechanisms driving astrocyte diversity.

## Physiology

As astrocytes were generally considered to be electrically non-excitable, the early discovery that they elevate their intracellular free Ca^2+^ levels in response to stimuli has attracted a lot of attention, as it suggests a means of cellular communication. Exploration of Ca^2+^ signaling has benefitted from the development of genetically encoded calcium indicators (GECIs), which can be targeted to various cellular locations (although live imaging is generally limited by the diffraction limit of fluorescence microscopy, meaning terminal leaflets are not visible). Use of these indicators has revealed a rich diversity of Ca^2+^ signals in cells, ranging from the generation of signaling microdomains [72,73], through to global waves that encompass entire astrocytes, including their cell bodies [74,75]. Ca^2+^ responses can occur spontaneously [73,76], or be evoked by neuronal activity [77,78].

The most frequent events are the formation of so-called ‘Ca^2+^ microdomains’ in astrocyte peripheral processes, which most likely occur due to the close physical proximity of processes to neurons and their exposure to neurotransmitters and/or neuromodulators [79]. There are likely to be multiple sources of Ca^2+^ within astrocyte processes. Ca^2+^ may enter directly in response to neuronal activity through the opening of ionotropic glutamate receptors, purinergic P_2_X receptors or nicotinic receptors. Alternatively, uptake of glutamate and γ-aminobutyric acid (GABA) leads to Na^+^ influx, which activates Na^+^/Ca^2+^ exchangers triggering Ca^2+^ transients. Activation of Gq-coupled metabotropic receptors leads to phospholipase C (PLC) activation and hydrolysis of phosphatidylinositol 4,5-bisphosphate (PIP_2_), resulting in the production of inositol 1,4,5-trisphosphate (IP_3_) (as well as diacylglycerol). High concentrations of cytoplasmic IP_3_ trigger Ca^2+^ release through type 2 IP_3_ receptors (IP_3_R_2_) in the endoplasmic reticulum: crucially, a local ER-derived Ca^2+^ signal may then propagate into a self-sustaining Ca^2+^ wave, due to Ca^2+^-induced Ca^2+^ release (CICR). Ca^2+^ release from mitochondria resident in astrocyte processes (opposite synapses) has also been reported (summarized in [11] and references therein). Such a complex and interlinked system likely explains why astrocytes are capable of responding to single action potentials with ‘microdomain’ events [78] and encoding stimuli of increasing intensity as a gradual accumulation and spread of Ca^2+^ along the process [77], with strong activity leading to global changes, which appear capable of inducing modifications in gene expression through cAMP response element binding protein (CREB) activation [27] (Figure 4C).

In hippocampal astrocytes, oscillations in intracellular Ca^2+^ appear superimposed on a heterogeneous resting Ca^2+^ landscape within the cell, with a gradient extending from the cell soma to the processes. This gradient is age-dependent and astrocytes with high and low Ca^2+^ occupy contiguous space [80]. The basal Ca^2+^ level is set by many factors, including Ca^2+^ leak from internal stores or through plasma membrane channels, the levels and distribution of fixed and mobile Ca^2+^ buffers and uptake/extrusion mechanisms [11]. Hence, heterogeneity in the basal Ca^2+^ reflects the Ca^2+^ homeostasis machinery at work in astrocytes (including presumably the peripheral processes), which will ‘sculpt’ the dynamics of evoked Ca^2+^ increases [81]. Variations in the cellular Ca^2+^ handling machinery presumably underlie the differences in spontaneous and evoked Ca^2+^ activities in hippocampal and striatal astrocytes [28] – and may go some way to explaining the differences in activation state evoked by Gi-coupled DREADDs (Designer Receptors Exclusively Activated by Designer Drugs) in these cells [28]. An additional level of complexity arises from the fact that astrocyte structure is known to be plastic and changes according to activity (see ‘Morphology’), meaning it is possible that Ca^2+^ signaling in a given cell may even change according to its previous activity.

Despite (these) major advances in our understanding of Ca^2+^ signal types, and the mechanisms underlying their generation, their physiological significance and information content are not well understood – although recent work suggests that reducing the amplitude and duration of Ca^2+^ signals in striatal astrocytes leads to excessive self-grooming in mice [82], while impairing Gi-coupled Ca^2+^ signaling brain wide leads to impaired spatial memory [83]. The introduction of next generation signal processing tools [84–86], capable of accurately describing the dynamics of (subcellular) Ca^2+^ transients, will undoubtedly help link the appearance of specific astrocytic Ca^2+^ signals to specific functional outputs. This will have to be allied to improvements in imaging to capture the true spatial dynamics of the signal (3D imaging) [87], over extreme time domains extending from milliseconds to seconds [88]. Major hurdles will still remain, however, to understand the molecular mechanisms linking a specific Ca^2+^ signal to its function output, such as control of local translation, local protein trafficking and protein complex formation, triggering of gliotransmitter release etc.

Crucially, a focus on intracellular Ca^2+^ elevations as activity signals may be unnecessarily constraining our view of astrocyte physiology, as dopamine appears to both elevate and lower astrocyte Ca^2+^ in the stratum radiatum [89], and it will be interesting to assess the functional consequences of such decreases. Beyond Ca^2+^, the use of genetically encoded indicators for cAMP [90] and lactate [91] has revealed transient changes in the intracellular concentrations of these molecules under defined conditions - and these signaling pathways have been reported to have crucial roles in astrocyte maturation and function [27]. In this respect, it is not surprising that neuronal activity appears to engage multiple second messenger pathways in a transmitter-specific, context dependent manner, providing yet another layer of complexity to astrocyte signaling and information processing [92].

Finally, although astrocytes were long assumed to be electrically passive cells, recent experiments with genetically encoded voltage sensors suggest that neuronal activity induces large, rapid, focal and pathway-specific depolarizations in astrocyte peripheral processes. These are driven primarily by action-potential mediated K^+^ efflux and the activity of electrogenic glutamate transporters [93]. These focal depolarizations inhibit astrocyte-mediated glutamate clearance during neuronal activity, enhancing glutamate receptor-mediated transmission in cortical pyramidal neurons. These findings further reinforce the view that astrocyte processes represent functionally independent units capable of influencing local neuronal activity. It is to be expected that local changes in membrane potential will exert powerful effects on astrocyte functions other than neurotransmitter clearance.

## Function

Perhaps the single most important advantage of astrocyte diversity is that it enables the creation of specialized neuron–glia units, which can drive complex behaviors. Strong evidence exists that specialist populations of spinal cord astrocytes influence local neural circuits. Astrocytes in the mouse ventral spinal cord express *Sema3a*, which is essential for axon integrity and survival of α-motor neurons during development [94]. Similarly, ventral horn astrocytes are also enriched in transcripts for *Kcnj10*, which encodes the astrocyte K^+^ channel Kir4.1. Interestingly, expression of Kir4.1 at sufficient levels was dependent on receiving glutamatergic input from local motor neurons, with loss of astrocyte Kir4.1 selectively impacting on the size of fast α-motor neurons and their function, implying a complicated functional feedback loop [95]. Evidence for astrocyte participation across a broad range of key biological functions has been obtained, including generation of circadian rhythms [96], respiration [97], food intake [98] and processing [99], and memory acquisition [100]. Recent work suggests that hippocampal astrocytes may even act as spatial processors, which encode reward location [101], although the encoding mechanism(s) used by the astrocyte and the consequences for local circuit activity are unknown at the time of writing.

Functional heterogeneity may not only be represented in a region-specific manner. Recent work supports the idea that astrocytes control cortical information processing: endocannabinoid release from cortical neurons leads to astrocyte activation and ATP/adenosine release, which acts to transiently depress synaptic transmission in both a column and layer-specific manner, consistent with a view of self-contained neuron-astrocyte signaling units in cortex (see also ‘Development’) [102]. Evidence for a similar degree of discrimination, based on endocannabinoid signaling, has also been found in the dorsal striatum, which contains intermingled subtypes of medium spiny neurons, that express either dopamine D1 or dopamine D2 receptors. When stimulated, endocannabinoid release from specific striatal neurons leads to increases in intracellular Ca^2+^ in different subsets of striatal astrocytes: crucially, mimicking this activation, using Ca^2+^ uncaging, evokes a synaptic potential only in homotypic pairs of synaptically connected neurons [103].

As astrocytes occupy unique, non-overlapping spatial positions in the parenchyma, it is likely that an individual astrocyte will contact 10,000s if not 100,000s of synapses [28,104,105]. Hence, from a pure spatial perspective, it is likely that astrocytes will contact both excitatory and inhibitory synapses. An interesting idea is that astrocyte peripheral process heterogeneity underlies differential synapse formation. For example, the expression of proteins such as neuroligin 2 and thrombospondin 1 may orchestrate excitatory synapse formation in one process [106], with inhibitory synapse formation relying on the specific expression and trafficking of proteins such as neuronal cell adhesion molecule 1 (NRCAM1) in a different process [107]. Alternatively, both sets of proteins may be expressed in discrete ‘signaling microdomains’ within the same process (Figure 4D). Furthermore, it is likely that the protein composition of astrocyte processes will dynamically change with functional consequences. For example, developmental upregulation of connexin 30 is essential for closing the critical period in mouse visual cortex [108], which it achieves by inhibiting expression of the matrix degrading enzyme matrix metallopeptidase 9 (MMP9), leading to stabilization of perineuronal nets, increased interneuron activity and circuit maturation.

Taken together, these examples provide clear proof that specialized astrocyte subsets impact the function of specific neural circuits and are likely capable of synapse-specific regulation. Unfortunately, at present our ability to identify unique astrocyte subtypes, usually by sequencing, has largely outstripped our ability to manipulate them functionally: given the complex transcriptional ‘fingerprints’ which increasingly define astrocyte subtypes, it is likely that intersectional genetic strategies will be needed for labeling [109]. Although new tools, such as adeno-associated virus (AAV)-based vectors, are simplifying genetic manipulations in mice [3], experiments in classical genetic models appears a promising parallel strategy (see also ’Transcriptome/Proteome’). For example, the power of genetics to help dissect the role(s) of glia in the control of circuit function was recently demonstrated in Zebrafish [110]. In response to failed swim attempts, sustained noradrenergic signaling led to the accumulation of Ca^2+^ in radial astrocytes, ultimately driving activity in GABAergic neurons to inhibit swimming – which presumably reflects an evolutionary constrained behavior to prevent unnecessary energy expenditure on futile tasks. In *Drosophila*, neurexin-neuroligin interactions were recently shown to close a critical period of motor circuit plasticity [111].

## Conclusions

Astrocyte heterogeneity is now an established concept. Aspects of development, molecular profile, intrinsic physiology and function all combine to produce a unique astrocyte identity in the adult (mouse) brain. Emerging evidence suggests that astrocytes show a high degree of plasticity, which allows them to adapt to the needs of local neurons and circuits. This heterogeneity may even exist at the level of individual peripheral processes, and may help fine tune synaptic transmission at individual synapses. However, a number of key questions remain unanswered. These include, but are not limited to, how is heterogeneity established and maintained at the level of individual processes, how is it modified over the complete range of synaptic/circuit activity and what is the potential influence of ageing and/or disease and injury?

## Summary

- Astrocytes show heterogeneity in developmental origin, mature morphology, molecular profile, intrinsic physiology and functional output.
- Heterogeneity occurs not only between brain regions but also within brain regions.
- Heterogeneity likely underpins the formation of specific astrocyte-neuron interactions essential for the formation of the specialized neural circuits that drive complex behaviors.
- Mounting evidence suggests that heterogeneity extends to the subcellular level, with astrocyte peripheral processes acting as local signaling domains capable of responding to, and influencing, local neuronal activity.
- New tools and experimental strategies – such as live super-resolution imaging combined with genetic methodologies allowing real-time monitoring of mRNA and local protein trafficking, or fluorescent probes to monitor astrocyte and neuron structure and function – are needed to understand the exact range of astrocyte heterogeneity and its functional consequences. In this respect, the more extensive use of genetic model organisms (*C. elegans, Drosophila*, Zebrafish) offers great potential to drive advances in the field.

## Abbreviations

CNS: Central nervous system
GFAP: Glial fibrially acidic protein
EAAT1: Excitatory amino acid transporter 1/GLAST
GFP: Green fluorescent protein
GPCR: G-protein coupled receptor
IP_3_R_2_: Type 2 inositol 1,4,5-trisphosphate receptor
MCU: Mitochondrial Ca^2+^ uniporter
(m)NCX: (Mitochondrial) Na^+^/Ca^2+^ exchanger
mPTP: Mitochondrial permeability transition pore
PMCA: Plasma membrane Ca^2+^ ATPase
SERCA: Sarcoplasmic/endoplasmic reticulum Ca^2+^ ATPase
α2δ-1: Voltage-dependent calcium channel subunit α2δ-1
NL: Neuroligin
NRCAM: Neuronal cell adhesion molecule
NRX: Neurexin
TGF-β1: Transforming growth factor-β1
TSP: Thrombospondin
DG: Dentate gyrus
STED: Stimulated emission depletion
FRET: Förster resonance energy transfer
TRAP: Translating ribosome affinity purification
EAAT2: Excitatory amino acid transporter 2/GLT1
smFISH: Single molecule fluorescence *in situ* hybridization
SHH: Sonic hedgehog
FGF: Fibroblast growth factor
GECI: Genetically encoded Ca^2+^ indicator
GABA: γ-aminobutyric acid
PLC: Phospholipase C
PIP_2_: Phosphatidylinositol 4,5-bisphosphate
IP_3_: Inositol 1,4,5-trisphosphate
CICR: Ca^2+^-induced Ca^2+^ release
CREB: cAMP response element binding protein
DREADD: Designer receptor exclusively activated by designer drugs
MMP9: Matrix metallopeptidase 9
AAV: Adeno-associated virus
